# Selenium inhibits ferroptosis in hyperglycemic cerebral ischemia/reperfusion injury by stimulating the Hippo pathway

**DOI:** 10.1371/journal.pone.0291192

**Published:** 2023-09-08

**Authors:** Lu Li, Meng Wang, Yan-Mei Ma, Lan Yang, Deng-Hai Zhang, Feng-Ying Guo, Li Jing, Jian-Zhong Zhang

**Affiliations:** 1 Ningxia Key Laboratory of Craniocerebral Diseases, Department of Pathology, School of Basic Medical Science, Ningxia Medical University, Yinchuan, Ningxia, China; 2 The Shanghai Health Commission Key Lab of AI-Based Management of Inflammation and Chronic Diseases, The Gongli Hospital of Shanghai Pudong New Area, Shanghai, China; Helwan University, EGYPT

## Abstract

Hyperglycemia can exacerbate cerebral ischemia/reperfusion (I/R) injury, and the mechanism involves oxidative stress, apoptosis, autophagy and mitochondrial function. Our previous research showed that selenium (Se) could alleviate this injury. The aim of this study was to examine how selenium alleviates hyperglycemia-mediated exacerbation of cerebral I/R injury by regulating ferroptosis. Middle cerebral artery occlusion (MCAO) and reperfusion models were established in rats under hyperglycemic conditions. An in vitro model of hyperglycemic cerebral I/R injury was created with oxygen-glucose deprivation and reoxygenation (OGD/R) and high glucose was employed. The results showed that hyperglycemia exacerbated cerebral I/R injury, and sodium selenite pretreatment decreased infarct volume, edema and neuronal damage in the cortical penumbra. Moreover, sodium selenite pretreatment increased the survival rate of HT22 cells under OGD/R and high glucose conditions. Pretreatment with sodium selenite reduced the hyperglycemia mediated enhancement of ferroptosis. Furthermore, we observed that pretreatment with sodium selenite increased YAP and TAZ levels in the cytoplasm while decreasing YAP and TAZ levels in the nucleus. The Hippo pathway inhibitor XMU-MP-1 eliminated the inhibitory effect of sodium selenite on ferroptosis. The findings suggest that pretreatment with sodium selenite can regulate ferroptosis by activating the Hippo pathway, and minimize hyperglycemia-mediated exacerbation of cerebral I/R injury.

## Introduction

Worldwide, stroke is a leading cause of mortality and disability [1, 2]. Patients with hyperglycemia are considered to be at higher risk of developing ischemic stroke and have a worse prognosis after ischemic stroke [3, 4]. Studies have suggested that drugs for cerebral I/R with normal blood glucose have poor effects on injury with hyperglycemia [5]. Previous studies have suggested that some natural plant extracts or trace elements, such as oxymatrine and gangliosides, have certain beneficial effects [6, 7]. The mechanisms are associated with antioxidant activity, reducing apoptosis and autophagy, and preserving mitochondrial function.

Ferroptosis is a type of regulated cell death that requires Fe^2+^ [8]. It has been suggested that ferroptosis is a type of neuronal injury after cerebral ischemia in addition to apoptosis and oxidative damage, and ferroptosis affects the recovery and prognosis of rats with cerebral ischemia [9]. Ferroptosis is distinct from apoptosis, necrosis, and autophagy [8]. Ferroptosis does not have characteristics of nuclear condensation and cell swelling. Ferroptosis exhibits several unique features, including increased mitochondrial membrane density and cristae breakdown [10]. Glutathione (GSH) depletion and glutathione peroxidase 4 (GPX4) or system Xc^-^ inactivation caused by catalysis of Fe^2+^ result in ferroptosis [11, 12], which leads to ROS accumulation and cell death. Previous reports have shown that a reduction in lactoferrin could aggravate ferroptosis in hyperglycemic mice after intracerebral cerebral hemorrhage [13].

Selenium can alleviate hyperglycemia-mediated exacerbation of cerebral I/R impairment [14–16], but the mechanism, and its impact on ferroptosis remains unknown. Determining how selenium affects ferroptosis will help us better understand how to treat cerebral ischemia damage that is aggravated by hyperglycemia and provide more comprehensive evidence for selenium treatment. Recent research has shown that activating the Hippo signaling pathway inhibits ferroptosis [17, 18]. Yes-associated protein (YAP) and transcriptional coactivator with PDZ-binding motif (TAZ) are effector molecules of the Hippo pathway [19]. Therefore, we hypothesized that sodium selenite could prevent hyperglycemia-mediated exacerbation of cerebral I/R impairment by attenuating ferroptosis through Hippo signaling pathway activation.

We conducted a series of in vitro and in vivo experiments to observe the therapeutic effects of sodium selenite and its effect on ferroptosis. In addition, since the Hippo pathway is associated with ferroptosis induced by cerebral ischemia injury, we observed how sodium selenite treatment affected the Hippo pathway. Hyperglycemic rats with middle cerebral artery occlusion (MCAO)were pretreated with sodium selenite before I/R, and oxygen-glucose deprivation and reoxygenation (OGD/R) plus high- glucose-induced cells were treated with sodium selenite (100 nM). Infarct volume, brain histopathological changes in the cortical penumbra region, Fe^2+^ content, the levels of GSH, MDA and SOD, cell viability, LDH release, and the expression of GPX4, YAP and TAZ were observed.

## Materials and methods

### Animals

Ten-week-old SD male rats were provided by Ningxia Medical University Laboratory Animal Center. The rats were randomly divided into four groups: the sham group (Sham), normoglycemic ischemia/reperfusion group (NG), hyperglycemic ischemia/reperfusion group (HG), and hyperglycemic ischemia/reperfusion pretreated with sodium selenite group (Se). Intraperitoneal injection of freshly dissolved streptozotocin (STZ, 60 mg/kg) in 0.1 M citric acid buffered saline was used to induce hyperglycemia (pH 4.5). Three days after STZ injection, blood glucose was tested. If the blood glucose level was higher than 16.7 mmol/L, the animals were included in the HG group. After the success of the hyperglycemic model, the Se group was dissolved in PBS with sodium selenite, and the rats were intraperitoneally injected with 0.4 mg/kg/day for 4 weeks. The Institutional Animal Care and Use Committee of Ningxia Medical University approved the procedures (no. IACUC‑NYLAC‑2019‑064), which were carried out strictly in compliance with the Chinese Laboratory Animal Use Regulation.

### Reagents

All reagents used in this study were purchased from relevant companies. The main reagents included STZ (S0130, Sigma, USA), sodium selenite (S5261, Sigma, USA), 2,3,5-triphenyltetrazolium chloride (TTC, T8877, Sigma, USA), Nissl staining assay kit (G1430, Solarbio, China), MDA, SOD and total GSH assay kits (#S0131M, #S0101S, #S0053, Beyotime Institute of Biotechnology, China), cell-counting kit-8 assay (CK04, Dojindo, Japan), cytotoxicity LDH Assay Kit-WST (CK12, Dojindo, Japan), Fe^2+^ assay kit (E-BC-K881-M, Elabscience, China), the ROS fluorescent probe- dihydroethidium (DHE) (R001, Vigorous Biotechnology, Beijing), nuclear and cytoplasmic extraction reagents (#78835, Thermo Fisher, USA), anti-YAP (#14074, Cell Signaling, USA), anti-TAZ, anti-glutathione peroxidase 4 (GPX4), anti-beta actin (ab224239, ab125066, ab8224, Abcam, China), goat anti-rabbit IgG and goat anti-mouse IgG (SA00013-4, SA00013-1, Proteintech, China).

### Middle cerebral artery occlusion (MCAO)

The rats were anesthetized, and the left common carotid artery (CCA), external carotid artery (ECA), and internal carotid artery (ICA) were exposed. The middle cerebral artery was blocked by inserting a silicone-coated filament via the ECA and moving it forward through the ICA until resistance was sensed. The filament was removes after 30 minutes in to restart blood flow. The sham group was subjected to the same treatment but without insertion of the filament.

### Assessment of infarct volume

Infarct volume was measured using 2,3,5-triphenyltetrazolium chloride (TTC) staining. The brain was cut into 5 slices and incubated in 1% TTC in the dark for 20 min at 37°C. The slices were placed in 4% paraformaldehyde overnight. Infarcted tissue was white, whereas noninfarcted areas appeared red. The infarcted area was measured by ImageJ and calculated under blinded conditions.

### Hematoxylin & eosin (H&E) and Nissl staining

H&E staining and Nissl staining were performed using standard protocols. Then, we counted the percentage of pyknotic neurons among total neurons and determined the mean optical density of Nissl bodies to assess neuronal damage.

### Transmission electron microscopy

The ischemic penumbra of the cortex was divided into 1 mm × 1 mm × 1 mm pieces. The tissue was placed in 2.5% glutaraldehyde overnight at 4°C and immersed in 1% osmic acid for two hours the following day. Then, the sample was dehydrated, embedded, sectioned, stained and examined. Mitochondrial length and the percentage of damaged mitochondria were measured by ImageJ to assess the extent of ferroptosis.

### Assessment of Fe^2+^ content

Brain tissues were cut and washed with PBS. Fe^2+^ content in brain tissues and HT22 cells were tested using an Fe^2+^ assay kit as directed by the manufacturer.

### Assessment of malondialdehyde (MDA), superoxide dismutase (SOD) and glutathione (GSH) levels

MDA, SOD, and total GSH levels in brain tissue were measured using the appropriate assay kits after the respective treatments.

### Cell culture and treatments

Cells were kept in DMEM/F12 with 10% fetal bovine serum and 1% penicillin/streptomycin at 37°C in a humidified incubator with 5% CO_2_. Using the OGD/R model in cells, we were able to simulate ischemia and reperfusion in vitro. The medium was changed to high glucose medium (50 mM) for 24 h, and sodium selenite was added to cells in the drug treatment group. Then, the cells were transferred to glucose-free medium and placed in a hypoxic environment with 1% O2, 5% CO2 and 94% N2 at 37°C (Thermo Scientific Series II, Merck). After 1 hour of OGD, the cells were switched to different culture media according to the experimental groups and placed under normal conditions for 24 hours. The cells were collected for future experiments.

### Cell viability assay

Cells were treated with CCK-8 solution for 20 minutes and measured at 450 nm with a microplate reader. LDH release was quantified according to the manufacturer’s recommendations. After the stepwise addition of each component, the absorbance of the samples was measured at 490 nm using a microplate reader.

### ROS fluorescent probe- (DHE)

The cells were treated with the ROS fluorescent probe- dihydroethidium (DHE, 2μM) for 30 minutes at 37°Cin the dark. Then, the cells were washed with fresh medium and viewed under a fluorescence microscope.

### Western blot analysis

Western blotting was performed as previously described [6]. The nuclear and cytoplasmic fractions of cells were separated according to the manufacturer’s instructions. Equal amounts of samples (30 μg protein/lane) were run separated on a 10% SDS‒PAGE gel. The proteins were transferred to a PVDF membrane, blocked with 5% nonfat milk for 2 hours at room temperature, and then incubated with the indicated antibodies. The following primary antibodies were used: anti-glutathione peroxidase 4 (1:1000), anti-ACSL4 (1:10000), anti-Ferritin (1:1000), anti-Ptgs2 (1:1000) and anti-beta actin (1:1000). After being washed, the membranes were treated for 1 h with appropriate secondary antibodies (goat anti-rabbit IgG, 1:5000 or goat anti-mouse IgG, 1:5000) at room temperature. Then, an ECL detection kit was used, and signals were imaged using a gel imaging system. ImageJ software was used to perform densitometric analysis of the bands, and the results were normalized to β-actin protein levels.

### Immunofluorescence analysis

Paraffin sections were dewaxed and rehydrated. The sodium citrate-based, heat-induced antigen retrieval method was used. Then, the slides were blocked with 10% goat serum for 30 minutes at 37°C after being permeabilized with 0.5% Triton X-100. The slices were exposed to the corresponding antibodies overnight at 4°C. The following primary antibodies were used: anti-GPX4 (1:200), anti-ACSL4 (1:200), anti-Ferritin (1:100), anti-Ptgs2 (1:50), anti-NeuN (1:100), anti-YAP (1:100) and anti-TAZ (1:100). Next, we rinsed and incubated the samples with secondary antibodies at 37°C in the dark for 1 hour. The slides were imaged using fluorescence microscopy.

### Statistical analysis

GraphPad Prism 8 and SPSS 25.0 were used to evaluate the data, which are shown as the mean ± SEM. We used a two-sample t test for independent samples or one-way analysis of variance (ANOVA) for multiple samples. When the P value was less than 0.05, differences were deemed statistically significant.

## Results

### Selenium reduced the infarct volume in vivo and cell injury in vitro

**Fig 1:**
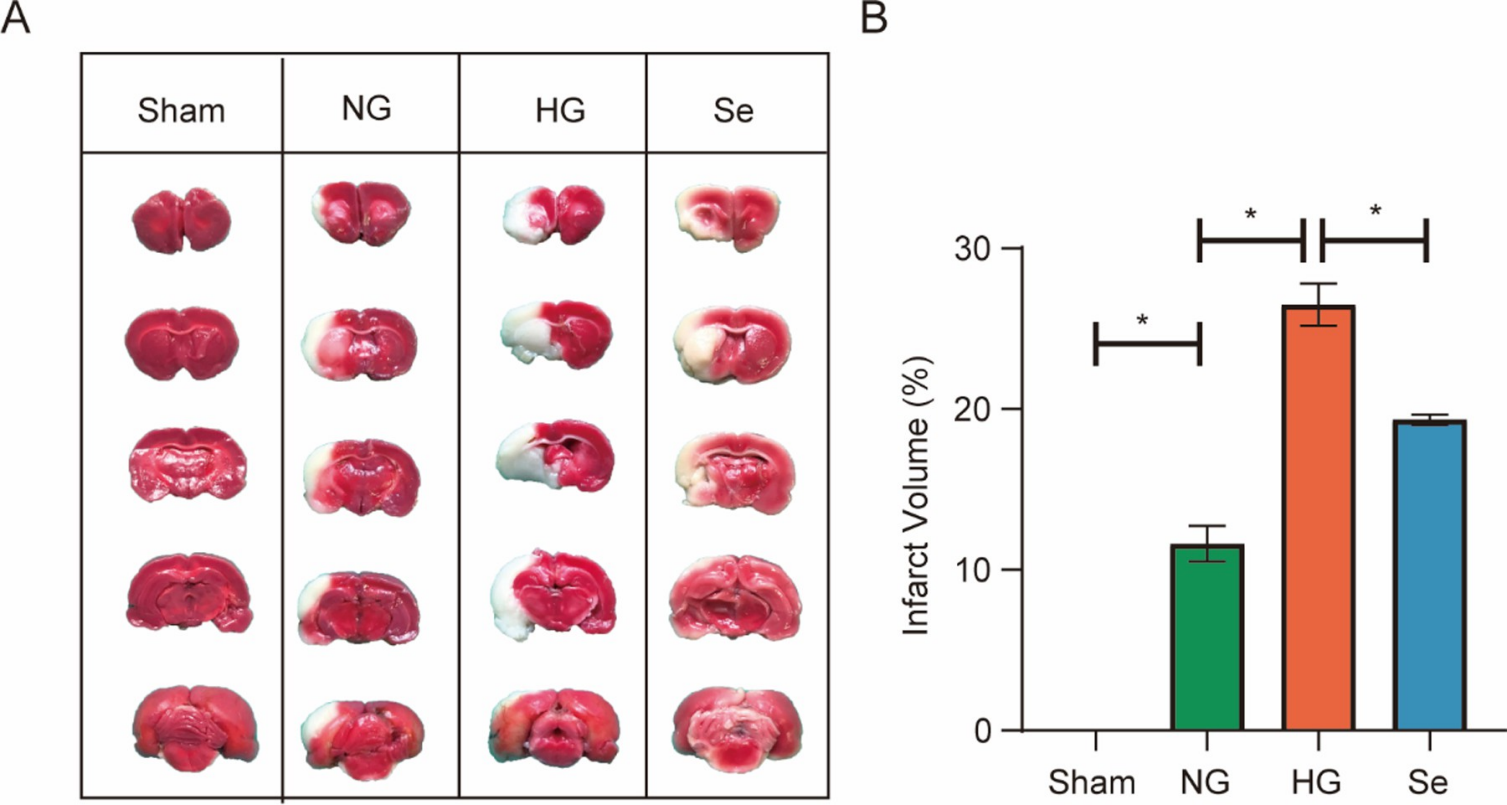
Infarct volume. (A) Cerebral infarct, as shown by TTC staining (n = 3). (B) Bar graph of infarct volume. ^*^P < 0.05.

As shown in Fig 1, TTC staining revealed a larger infarct volume in the HG group than in the NG group. Sodium selenite pretreatment reduced the infarct volume compared to HG (Fig 1A and 1B).

**Fig 2:**
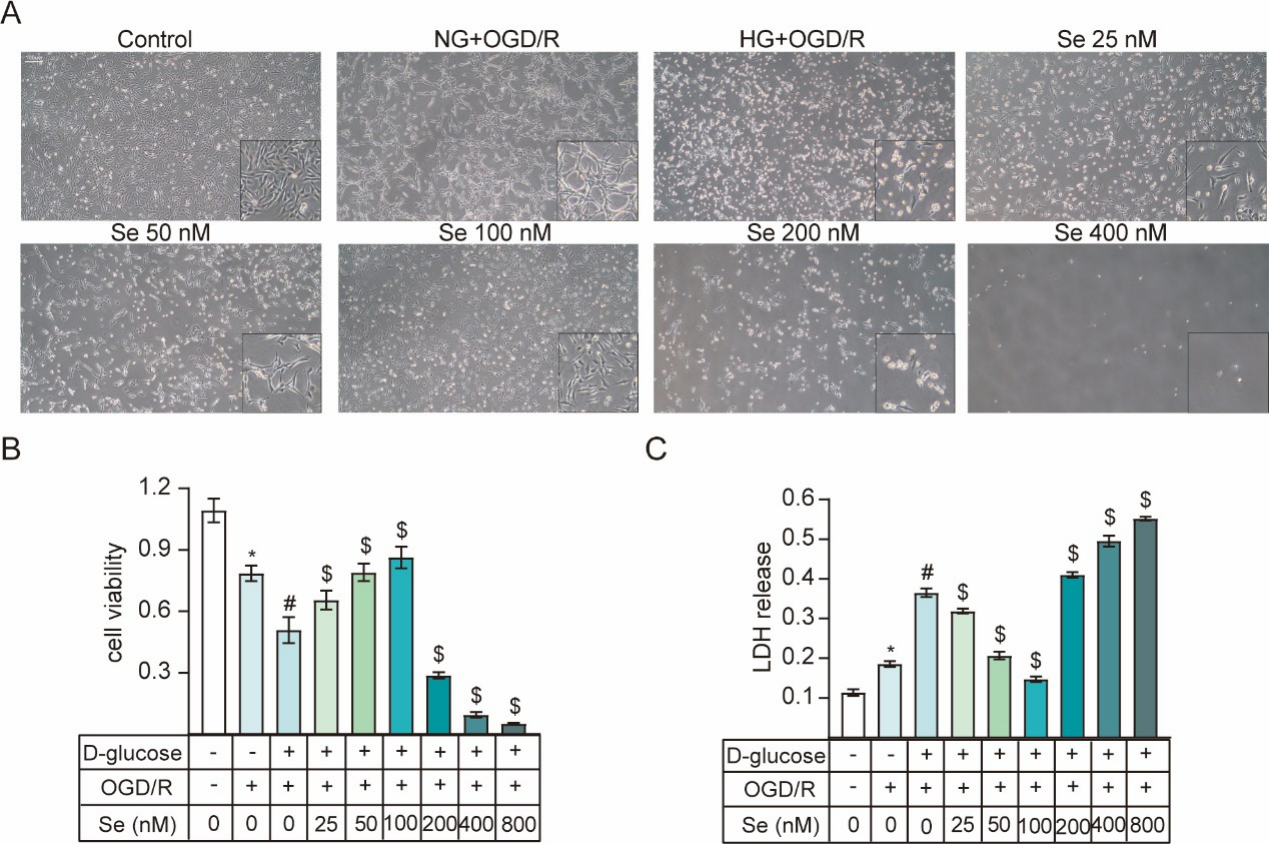
HT22 viability and LDH release. (A) HT22 cells (bar = 100 μm). (B) Cell viability (n = 5). (C) LDH release (n = 5). ^*^P < 0.05 *vs*. control. ^#^*P* < 0.05 *vs*. NG+OGD/R. ^$^*P* < 0.05 *vs*. HG+OGD/R.

The cells were treated with gradient concentrations of sodium selenite in OGD/R and high glucose conditions to study the impact of selenium. Fusiform cells with spindly pseudopods were present in the control group (Fig 2A). According to the CCK-8 and LDH release assays, cell viability decreased after OGD/R, while LDH release clearly increased. Cell viability decreased and LDH increased more significantly in the HG group than the NG group. Treatment with 6 concentrations of sodium selenite revealed that the degree of the increase in cell viability and reduction in LDH was the most obvious in response to 100 nM (Fig 2B and 2C).

### Selenium protected the brain and neurons after I/R plus hyperglycemia

**Fig 3:**
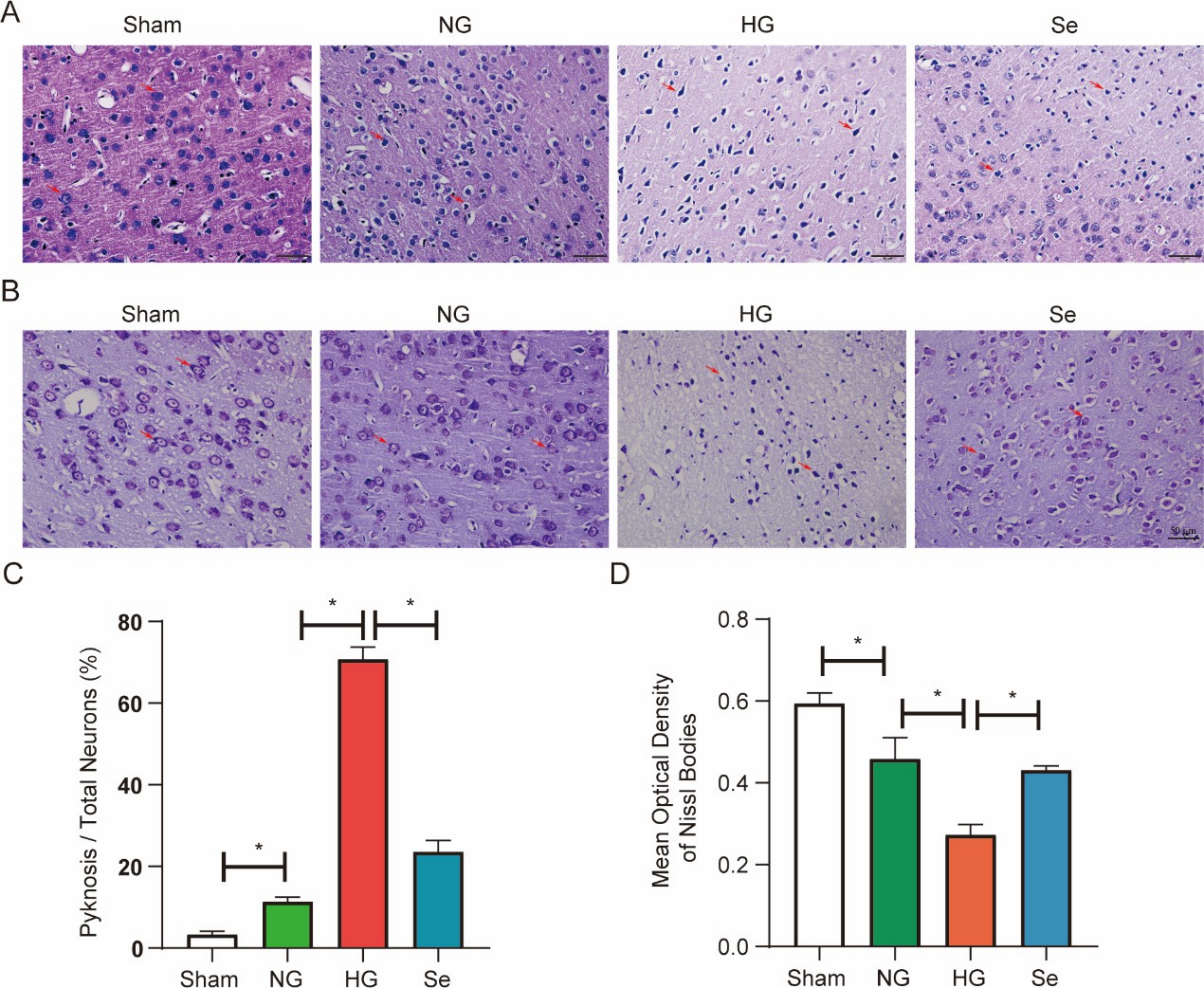
Selenium protects the brain and neurons in the penumbra. (A) Representative micrographs of H&E staining (bar = 50 μm). (B) Representative micrographs of Nissl staining (bar = 50 μm). (C) The ratio of pyknotic neurons (n = 3). (D) The mean optical density of Nissl staining (n = 3). ^*^P < 0.05.

In the NG group, edema and neuronal shrinkage increased significantly, while the optical density of Nissl staining decreased significantly (Fig 3). The optical density of Nissl staining was considerably lower in the HG group than in the NG group, and edema and shrinkage were significantly higher increased. (Fig 3). After pretreatment with sodium selenite, the degree of the increase in neuronal shrinkage was considerably lower than that in the HG group, whereas the optical density of Nissl staining was much higher than that in the HG group (Fig 3).

**Fig 4:**
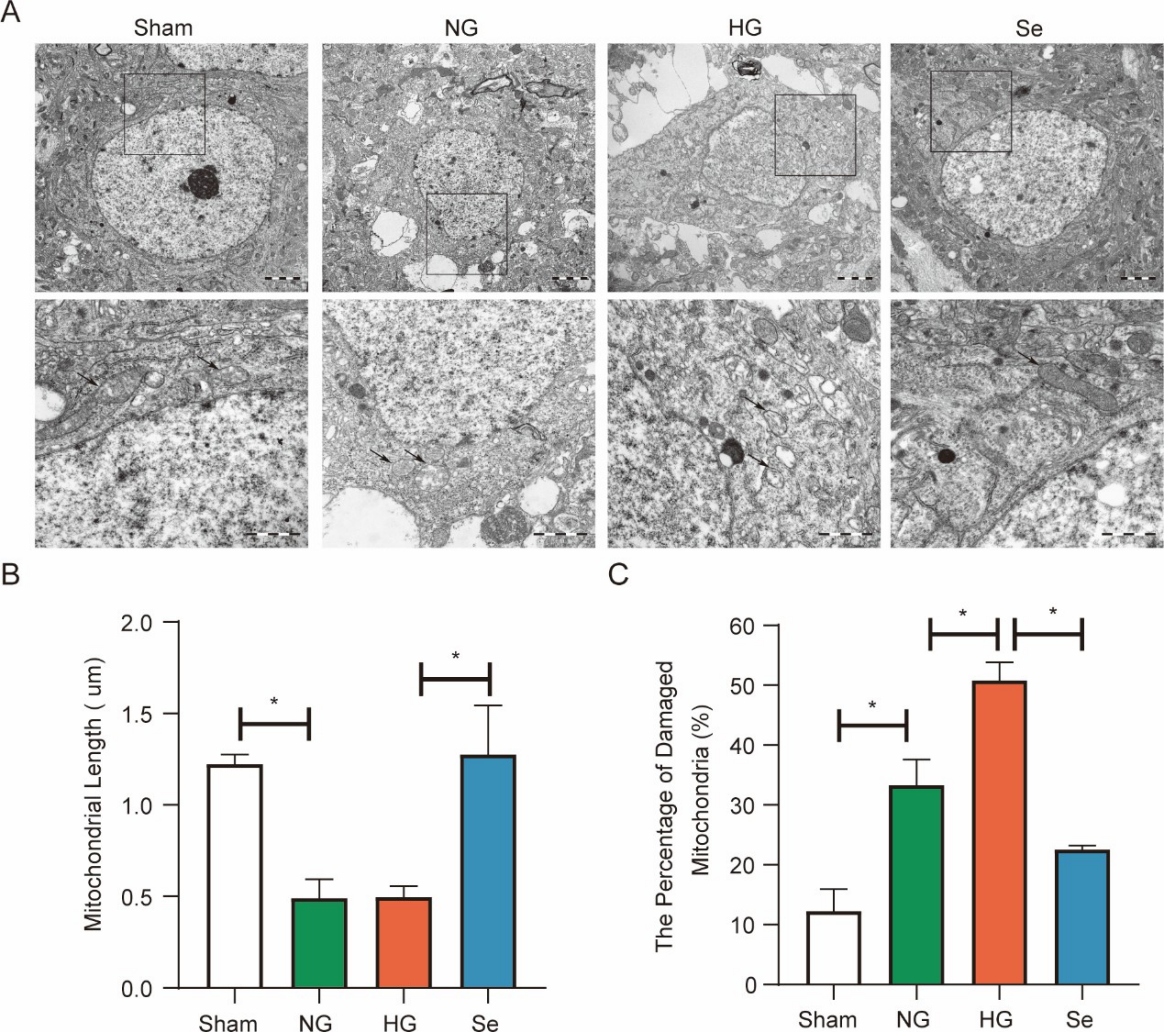
The ultrastructure of neurons in the penumbra. (A) Representative TEM images showing increased mitochondrial membrane density and broken cristae (arrow) (bar = 1 μm). (B) Bar graph of mitochondrial length (n = 3). (C) The percentage of damaged mitochondria (n = 3). ^*^P < 0.05.

The ultrastructural features of ferroptosis, such as increased mitochondrial membrane density and the breakdown of cristae, were observed by transmission electron microscopy (TEM). Although there was no significant difference in mitochondrial length between the NG and HG groups, the proportion of damaged mitochondria in the HG group was significantly higher than that in the NG group (Fig 4). These changes represent some characteristics of ferroptosis, and sodium selenite pretreatment reversed these changes (Fig 4).

### Selenium attenuated ferroptosis after I/R plus hyperglycemia

**Fig 5:**
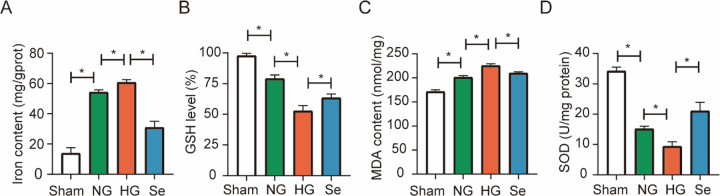
Bar graph of Fe^2+^, GSH, MDA and SOD levels in brain tissues. (A) Fe^2+^ (n = 3). (B) GSH (n = 3). (C) MDA (n = 3). (D) SOD (n = 3). ^*^ *P* < 0.05.

Fe^2+^ content in the brain were considerably increased after I/R (Fig 5A). Fe^2+^ content in the HG group were higher than those in the NG group (Fig 5A). Compared with that in the HG group, pretreatment with sodium selenite significantly decreased Fe^2+^ content (Fig 5A). GSH, which is an index of ferroptosis, was lower in the HG group than in the NG group, and pretreatment with sodium selenite increased GSH levels(Fig 5B). As shown in Fig 5C, MDA levels were considerably higher in the HG group than in the NG group, and sodium selenite treatment decreased MDA levels. The HG group had significantly lower SOD levels than the NG group, and sodium selenite increased the levels of SOD (Fig 5D).

**Fig 6:**
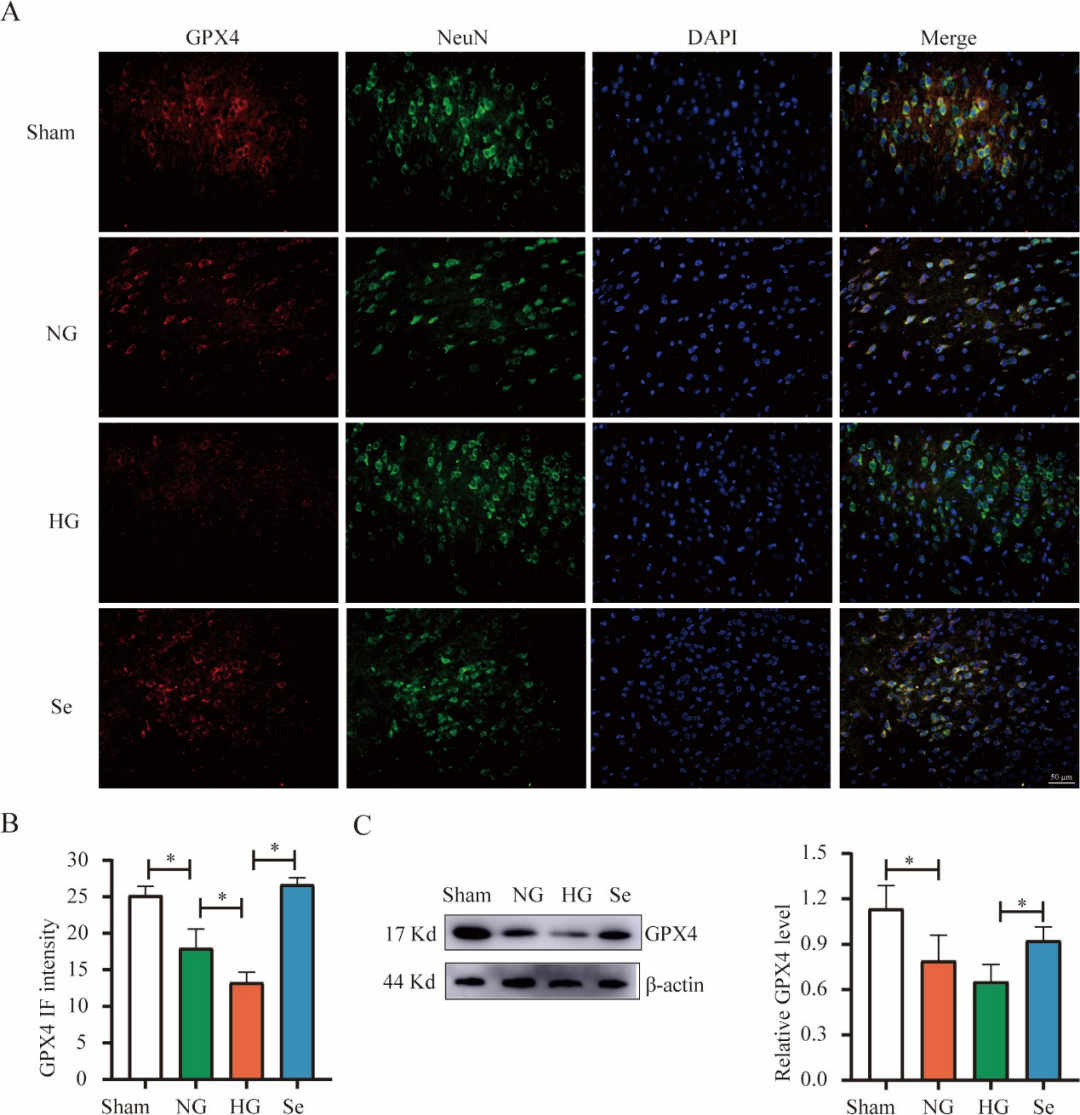
GPX4 protein in brain tissues. (A) Representative immunofluorescence images of GPX4 (red), NeuN (green) and DAPI (blue) (bar = 50 μm). (B) Bar graph of GPX4 fluorescence intensity in the whole field of view (n = 3). (C) Western blot analysis of GPX4 (n = 3). ^*^ *P* < 0.05.

GPX4, which is the primary marker of ferroptosis, was decreased in the HG group compared to the NG group and was significantly increased in rats that were pretreated with sodium selenite (Fig 6).

**Fig 7:**
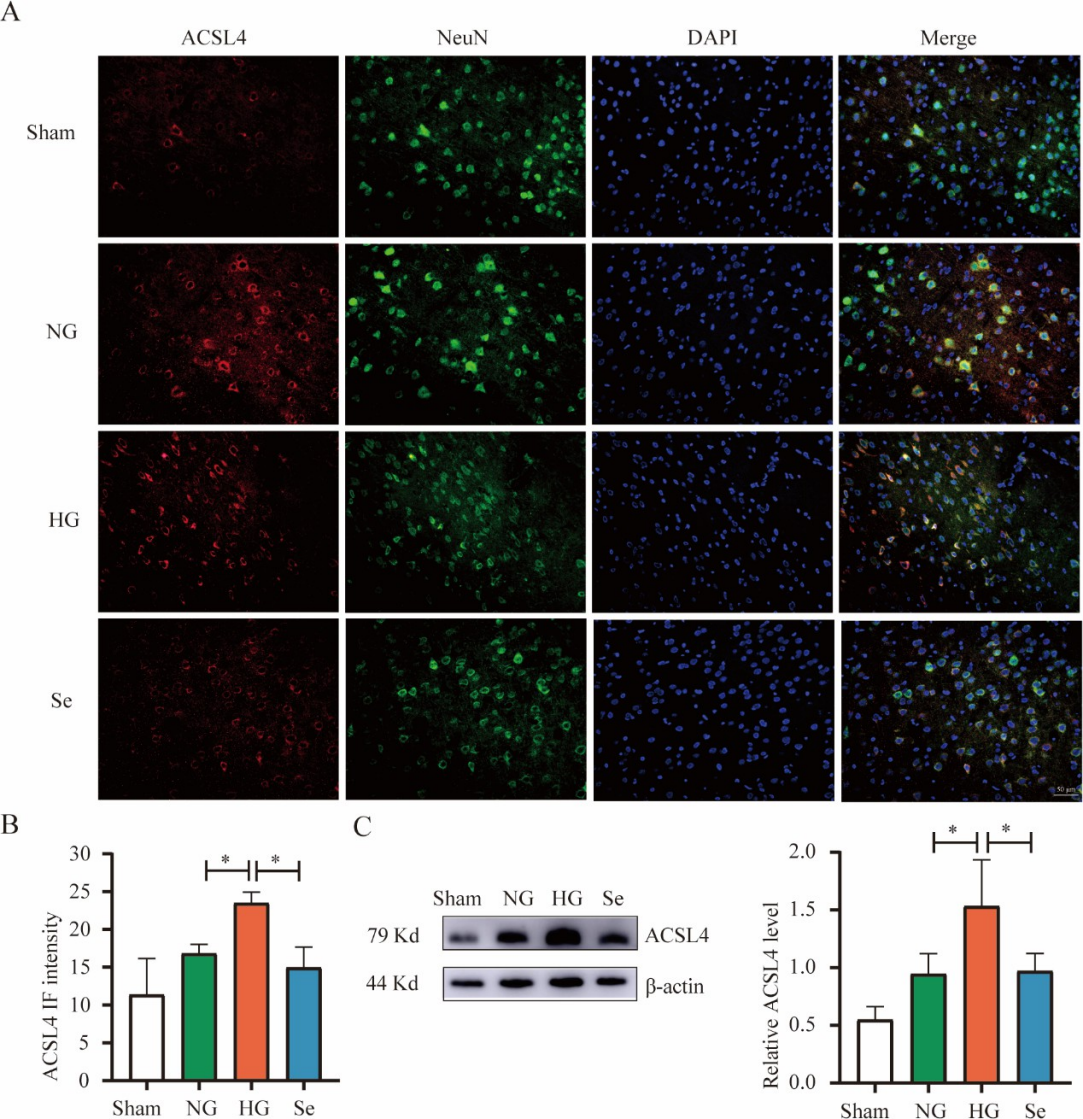
ACSL4 protein in brain tissues. **(**A) Representative immunofluorescence images of ACSL4 (red), NeuN (green) and DAPI (blue) (bar = 50 μm). (B) Bar graph of ACSL4 fluorescence intensity in the whole field of view (n = 3). (C) Western blot analysis of ACSL4 (n = 3). ^*^ *P* < 0.05.

ACSL4 is an important regulatory molecule in the ferroptosis and lipid metabolism pathways, and PUFAs can generate lipid hydroperoxides via ACSL4, thereby promoting the occurrence of ferroptosis. The NG group had a slightly higher ACSL4 level than the Sham group, but there was no significant difference. However, when compared to the NG group, the HG group had a large increase in ACSL4. When compared to the HG group, the Se treatment group had a substantial decrease in ACSL4 (Fig 7).

**Fig 8:**
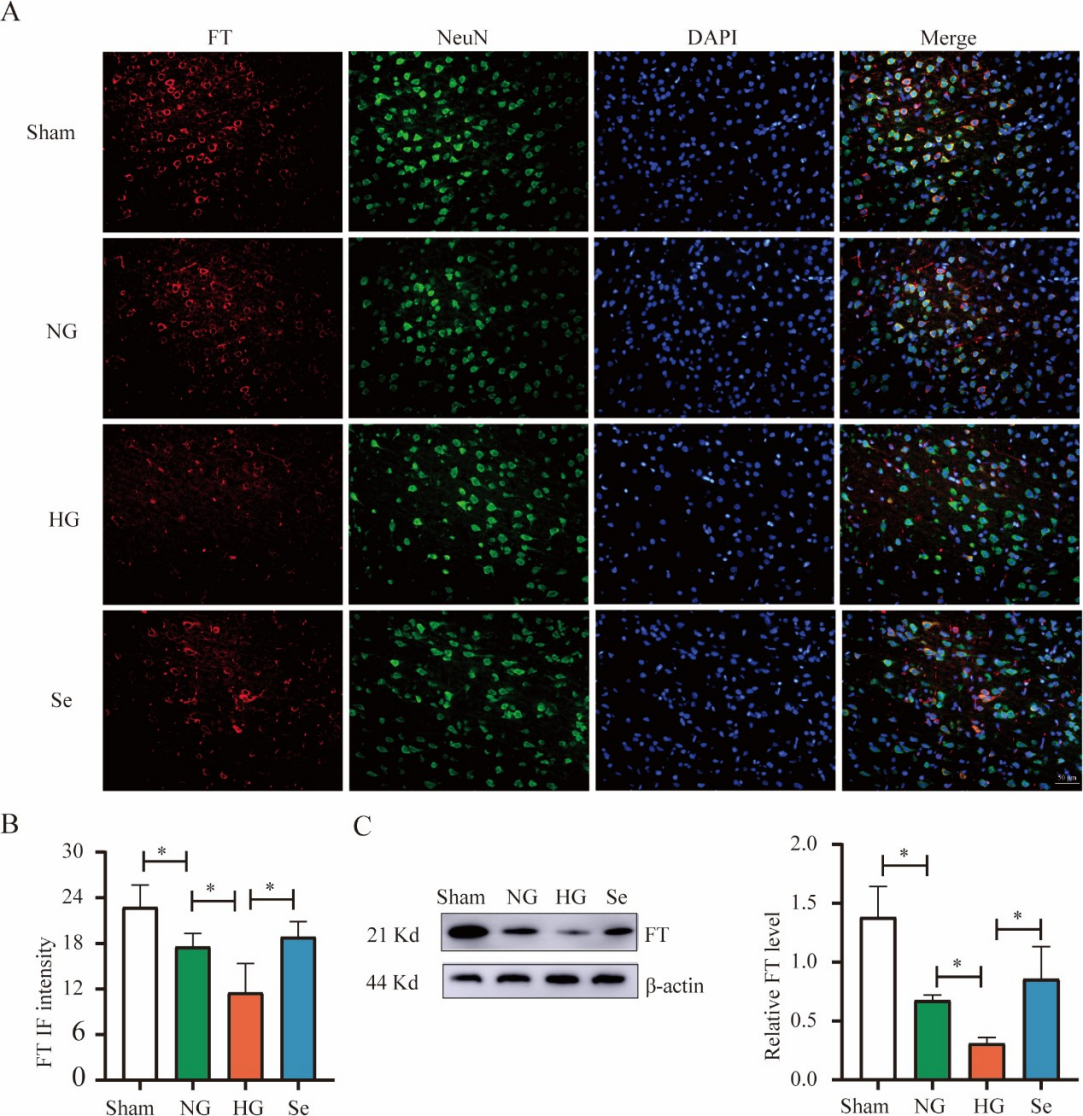
FT protein in brain tissues. (A) Representative immunofluorescence images of FT (red), NeuN (green) and DAPI (blue) (bar = 50 μm). (B) Bar graph of FT fluorescence intensity in the whole field of view (n = 3). (C) Western blot analysis of FT (n = 3). ^*^ *P* < 0.05.

FT can reduce intracellular iron levels by oxidizing Fe^2+^into Fe^3+^and storing it in ferritin, thereby reducing the sensitivity of cells to ferroptosis. The NG group showed a considerable decrease in FT compared to the Sham group, whereas the HG group showed a lagger decline than the NG group. When compared to the HG group, the Se group exhibited a significant increase in FT (Fig 8).

**Fig 9:**
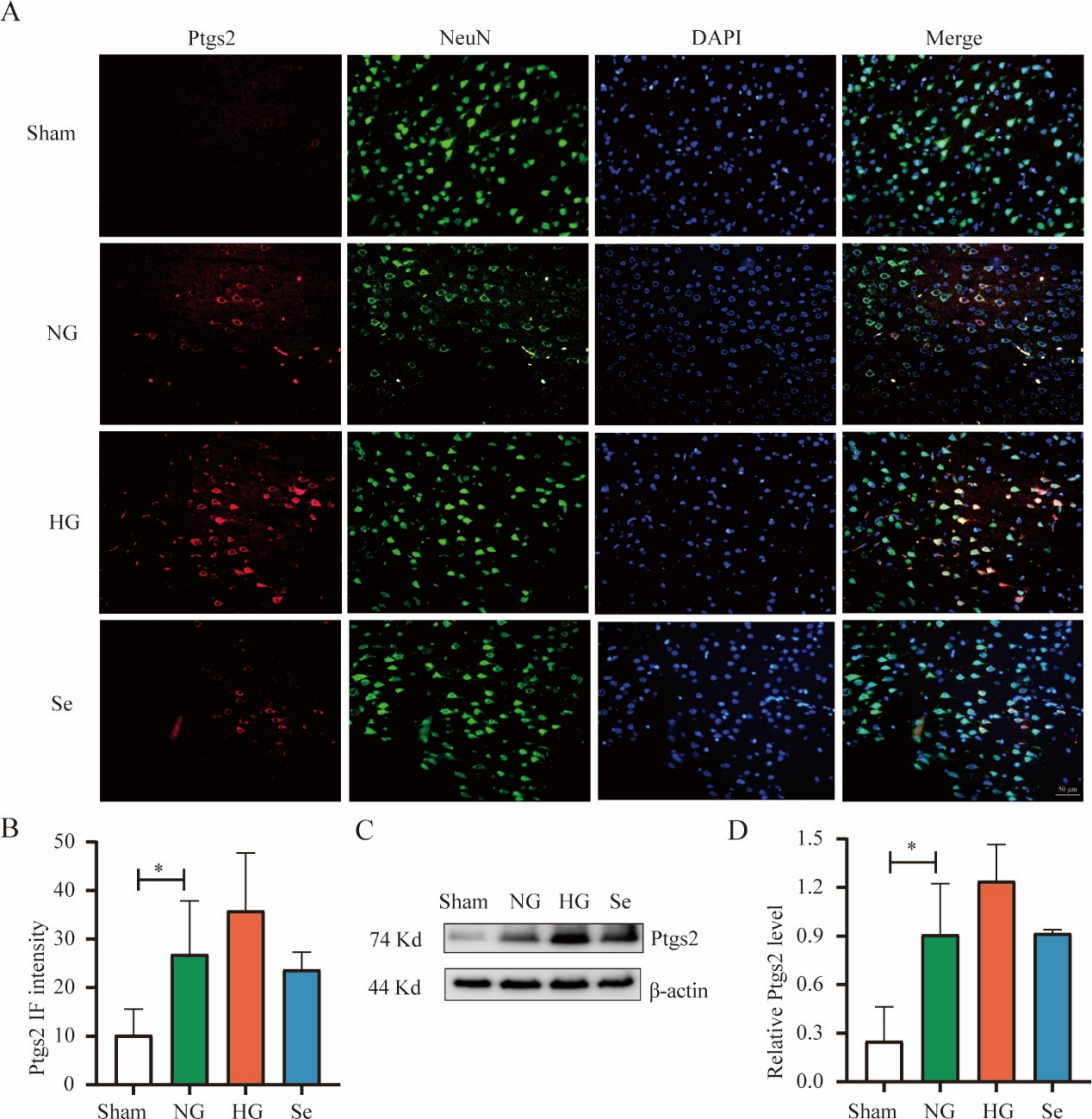
Ptgs2 protein in brain tissues. (A) Representative immunofluorescence images of Ptgs2 (red), NeuN (green) and DAPI (blue) (bar = 50 μm). (B) Bar graph of Ptgs2 fluorescence intensity in the whole field of view (n = 3). (C) Western blot analysis of Ptgs2 (n = 3). ^*^ *P* < 0.05.

Ptgs2 is now thought to be a biomarker of ferroptosis. After brain I/R, the NG group exhibited a substantial increase in Ptgs2 compared to the Sham group. The HG group showed an increase in Ptgs2 compared to the NG group, although there was no significant difference. Although Ptgs2 was decreased in the Se group, there was no significant difference (Fig 9).

**Fig 10:**
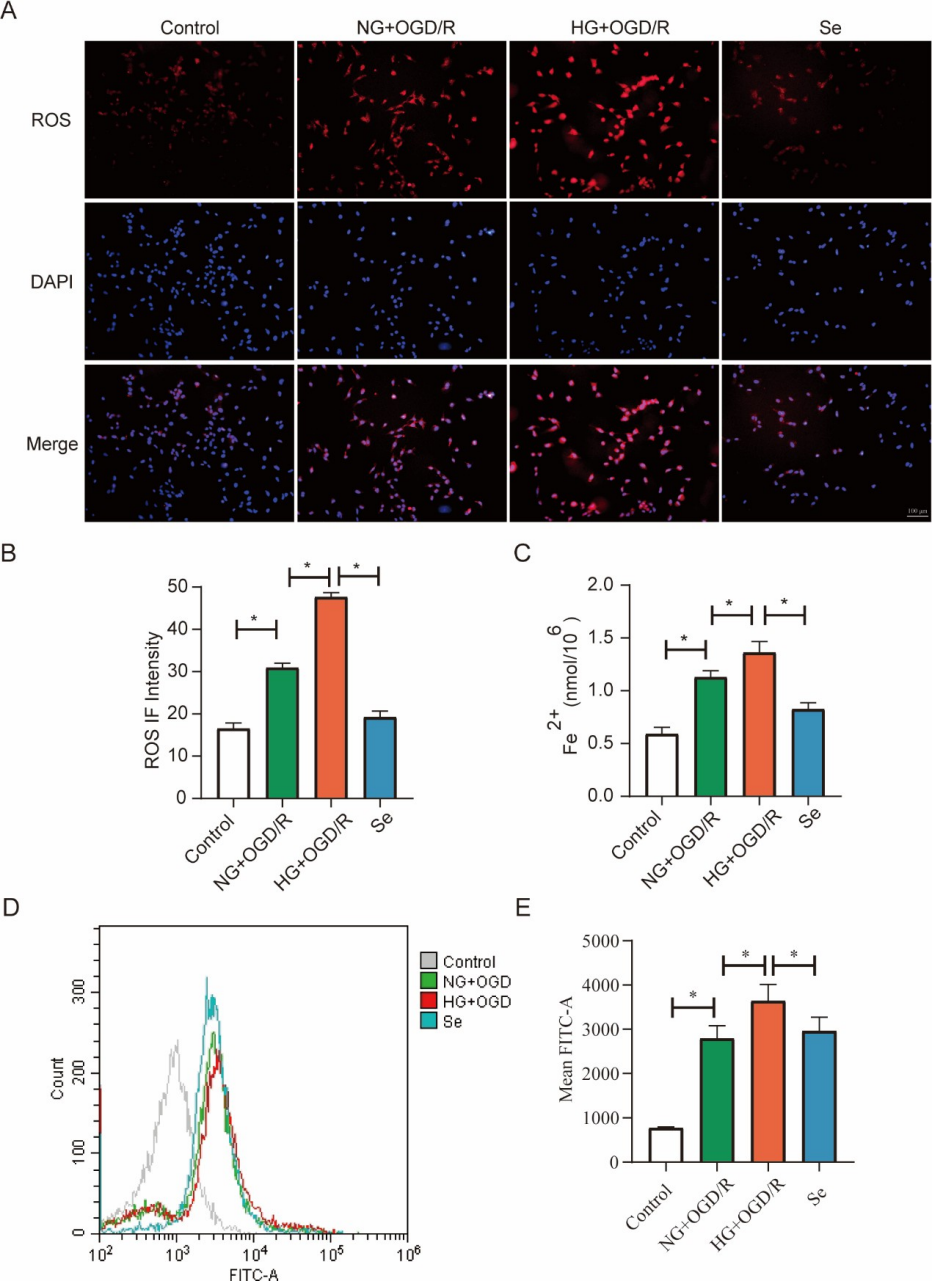
ROS and Fe^2+^ levels in HT22 cells. (A) Representative immunofluorescence images of ROS (red) and DAPI (blue) (bar = 100 μm). (B) Bar graph of ROS fluorescence intensity (n = 3). (C) Fe^2+^ content in HT22 cells (n = 3). (D) Representative images of the flow cytometry results. (E) Bar graph of the mean FITC-A fluorescence intensity. ^*^ *P* < 0.05.

In HT22 cells, ROS levels were increased in the HG group compared to the NG group, and pretreatment with sodium selenite prevented ROS production in the Se group (Fig 10A and 10B). The content of Fe^2+^ also showed similar results as the animal experiments (Fig 10C). In addition, we measured the degree of lipid peroxidation using a C11 BODIPY 581/591 fluorescent probe. The findings revealed that the lipid peroxidation levels in the NG+OGD/R group were significantly higher than those in the control group, and the HG group showed a lager increase than the NG group, while the Se pretreatment group showed a significantly lower level of lipid peroxidation than the HG group (Fig 10D and 10E).

### Selenium promoted GPX4 expression by activating the Hippo pathway

**Fig 11:**
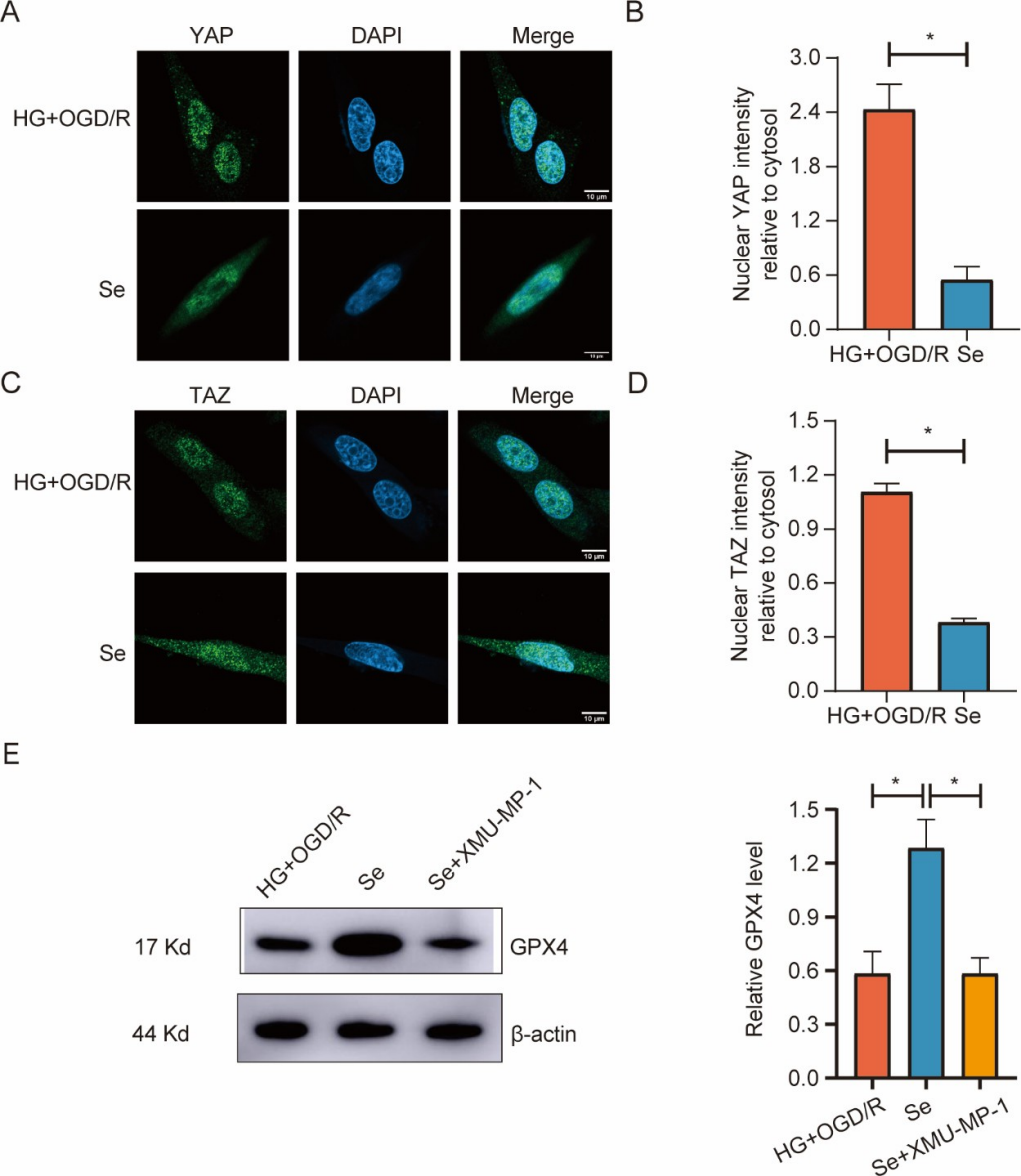
Expression of YAP, TAZ and GPX4 in HT22 cells. (A) Representative immunofluorescence images of YAP (green) and DAPI (blue) (bar = 10 μm). (B) Bar graph of the nuclear-cytoplasmic ratio of YAP. (C) Representative immunofluorescence images of TAZ (green) and DAPI (blue) (bar = 10 μm). (D) Bar graph of the nuclear-cytoplasmic ratio of TAZ. (E) Western blot analysis of GPX4. ^*^P < 0.05.

The results showed that the nuclear-cytoplasmic ratio of YAP and TAZ was considerably decreased in the Se group compared to the HG+OGD/R group (Fig 11A–11D). It was also shown that GPX4 expression was upregulated by selenium compared with HG+ OGD/R and XMU-MP-1 (an inhibitor of the Hippo signaling pathway) (Fig 11E).

**Fig 12:**
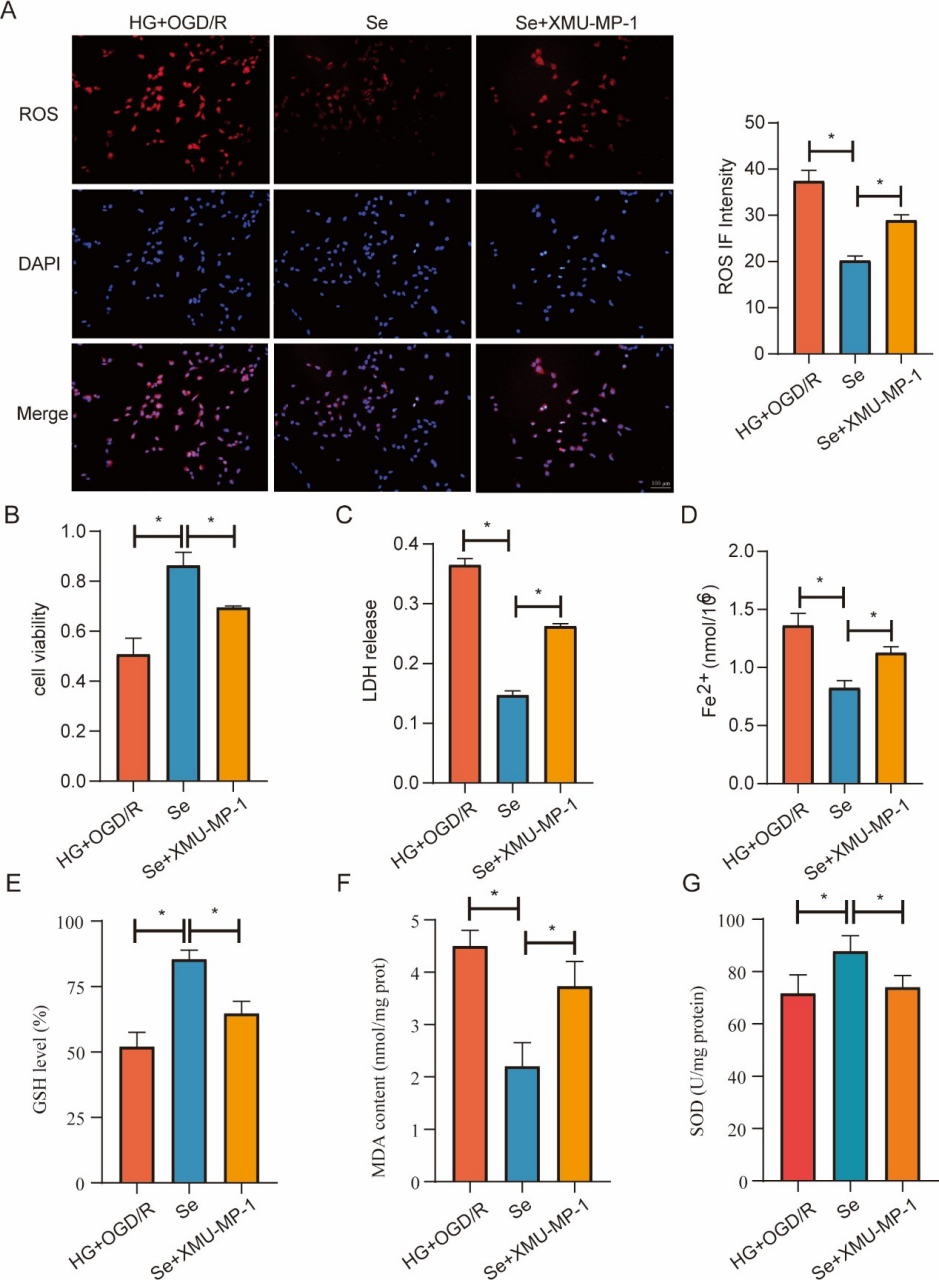
ROS levels, cell viability, and LDH and Fe^2+^ content in HT22 cells after XMU-MP-1 treatment. (A) Representative immunofluorescence images of ROS (red) and DAPI (blue) (bar = 100 μm) and the bar graph of the ROS fluorescence intensity (n = 3). (B) Bar graph of cell viability (n = 5). (C) Bar graph of LDH release (n = 5). (D) Bar graph of Fe^2+^ content (n = 5). (E) Bar graph of GSH levels (n = 3) F. Bar graph of MDA levels (n = 3). G. Bar graph of SOD levels (n = 3). ^*^P < 0.05.

This study showed that XMU-MP-1 greatly reduced cell activity and aggravated cell damage (Fig 12B), indicating that the protective effect of sodium selenite against high glucose OGD/R injury in cells was inhibited by XMU-MP-1. Additionally, we found that after adding XMU-MP-1, ROS levels increased (Fig 12A), LDH release increased (Fig 12C), Fe^2+^content significantly increased (Fig 12D), GSH levels decreased (Fig 12E), MDA levels increased (Fig 12F), and SOD activity decreased (Fig 12G). These results indicate that sodium selenite can inhibit ferroptosis, and that the inhibitory effect of sodium selenite on ferroptosis can be restored by XMU-MP-1. This findings suggests that sodium selenite works via the Hippo pathway.

## Discussion

According to research, hyperglycemia worsens brain injury after I/R, and research on corresponding drugs is still in progress [3, 4, 20]. However, finding a treatment for hyperglycemia-mediated exacerbation of cerebral I/R injury remains a major medical issue. Selenium (Se) is a nonmetallic chemical element and an essential nutrient for the human body [21]. Selenium can enhance human immunity. It is necessary to continuously supplement selenium from the diet to meet metabolic needs [22]. The physiological performance of several human organs and tissues is greatly aided by selenium concentrations. There are many diseases related to selenium deficiency, such as cardiovascular disease, immunological disease and cancer [23].

The findings of the current study revealed that rats had increased infarct volume, edema, and neuronal shrinkage in the penumbra of the cortex after ischemia/reperfusion and decreased Nissl staining in the penumbra of the brain in the HG group compared to the NG group. Compared with HG group, sodium selenite (0.4 mg/kg/day) injection for four weeks before MCAO could repair the injury indicated above. Moreover, pretreatment with sodium selenite (100 nM) maintained the vitality of HT22 cells after OGD/R. These results suggested that pretreatment with sodium selenite could alleviate I/R injury that is exacerbated by hyperglycemia, which is consistent with previous reports [24, 25].

The mechanism by which hyperglycemia exacerbates cerebral I/R injury may be associated with a variety of cellular injury pathways and extracellular signaling pathways [3, 26]. According to some research, hyperglycemia in mice greatly increases ferroptosis following stroke and contributes to increased brain damage [13, 27]. This study revealed that in hyperglycemic rats, in addition to the increase in infarct volume and histological damage, the density of the mitochondrial membrane increased and cristae decreased, as shown by TEM, Fe^2+^ content increased, the ferroptosis-related molecules GSH, SOD and GPX4 decreased, and MDA and ROS levels increased significantly compared with those in normoglycemic rats. After pretreatment with sodium selenite, mitochondrial structure was improved, GSH, SOD and GPX4 levels increased, and MDA and ROS levels decreased to some extent. Therefore, we proposed that hyperglycemia-mediated exacerbation of cerebral I/R injury was related to enhanced ferroptosis, and the alleviation of hyperglycemia- mediated exacerbation of cerebral I/R injury by sodium selenite may be linked to a decrease in ferroptosis. Ferroptosis is a type of regulated cell death that is dependent on iron [8], as opposed to apoptosis, necrosis and autophagy [28]. It has been reported that ferroptosis plays a role in neurotoxicity, neurodegenerative diseases, and T-cell immunity [29, 30].

Current evidence suggests that selenium is important for regulating ferroptosis [31, 32]. Some studies have suggested that Se has antioxidant and neuroprotective effects [33, 34]. Because ferroptosis is closely related to oxidative damage, Se can antagonize oxidation, thereby reducing ferroptosis [31, 35]. However, the mechanisms by which selenium reduces ferroptosis remain unclear, and it is possible that many pathways are involved [36, 37]. For example, recent studies have shown that selenium can prevent ferroptosis by controlling the Nrf2/GPX4 pathway in the BTBR mouse model of autism [36]. Other studies have shown that selenium can increase SLC7A11 levels and thereby reduce ferroptosis in aflatoxin B1-induced cardiotoxicity [37]. It was demonstrated in this study that compared that in to HG+ OGD/R cells, there was a decrease in the nuclear/cytosolic ratio of YAP and TAZ, as well as an increase in the expression of GPX4 in cells that were pretreated with sodium selenite. When cells were treated with XMU-MP-1, these results were similar to those in cells without sodium selenite pretreatment. These findings suggest that the suppression of ferroptosis by selenium may be involved in the Hippo signaling pathway, possibly because Se maintains the activity of the Hippo signaling pathway, promotes antioxidant effects, and thereby reduces ferroptosis. The Hippo signaling pathway is extremely well conserved, and its main members are YAP and TAZ, which play essential roles in the regulation of growth and apoptosis [38, 39]. The Hippo pathway contributes to the stability of the structure and function of the CNS. For example, YAP/TAZ is involved in the development and differentiation of neural precursor cells [40]. Furthermore, the Hippo pathway is important in maintaining the normal structure and function of components of the blood‒brain barrier (BBB) [41, 42]. The cell death mechanism mediated by the Hippo signaling pathway will help to identify effective treatment strategies for related diseases [43]. However, the specific mechanism by which the Hippo signaling pathway controls cell death is still unclear, and its role in ferroptosis during hyperglycemic cerebral I/R injury requires further study.

Our study focused on 24 hours after cerebral I/R while ignoring the complicated and constantly evolving pathophysiologic process of the disease. It remains to be seen whether these findings apply to other stages of cerebral I/R injury. In addition, only one the HT22 cell line, was selected for the in vitro experiments in this study, which has several limitations.

In conclusion, in the current study, the hyperglycemic rat model was pretreated with sodium selenite before MCAO and reperfusion, and high glucose and OGD/R-induced cells were treated with sodium selenite (100 nM). The results showed that hyperglycemia aggravated cerebral ischemia/reperfusion injury, and sodium selenite reduced the infarct volume, edema and neuronal shrinkage in the penumbra. Pretreatment with sodium selenite reduced the enhancement of ferroptosis related to hyperglycemia, which maintained of mitochondrial structure, decreased Fe^2+^, MAD and ROS levels, and increased GSH and GPX4levels. The immunofluorescence localization results showed that pretreatment with sodium selenite increased YAP and TAZ levels in the cytoplasm but decreased YAP and TAZ levels in the nucleus and maintained the expression of GPX4, which was inhibited by high glucose. XMU-MP-1 eliminated the effects of sodium selenite. These findings suggest that sodium selenite preconditioning could reduce cerebral injury induced by ischemia/reperfusion plus hyperglycemia, which is associated with the inhibition of ferroptosis and may be involved in the protection of the Hippo signaling pathway. Selenium may be an effective agent to treat cerebral ischemia/reperfusion under hyperglycemia.

## Supporting information

S1 Raw images (PDF)
