# Supplementary material for: Selenium inhibits ferroptosis in hyperglycemic cerebral ischemia/reperfusion injury by stimulating the Hippo pathway
Source: PLoS One. 2023 Sep 8;18(9):e0291192. doi: 10.1371/journal.pone.0291192 (PMC10490962; doi:10.1371/journal.pone.0291192)
Supplement: S1 Raw images — (PDF) [file pone.0291192.s001.pdf]

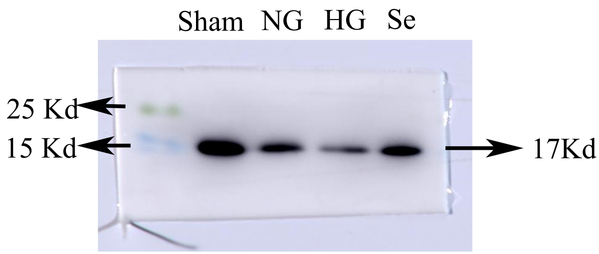

Fig 6. GPX4

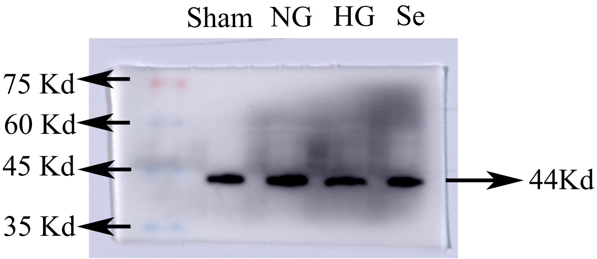

Fig 6. β- actin

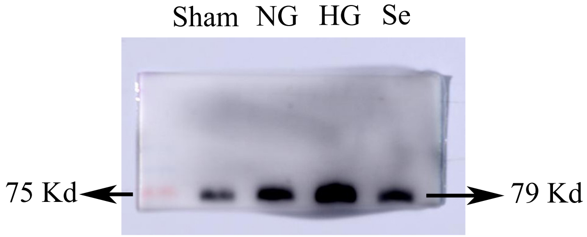

Fig 7. ACSL4

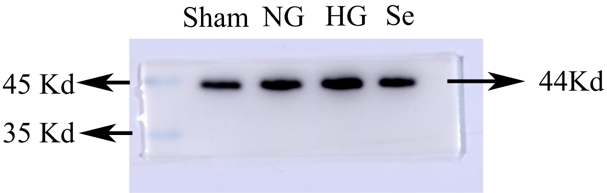

Fig 7. β- actin

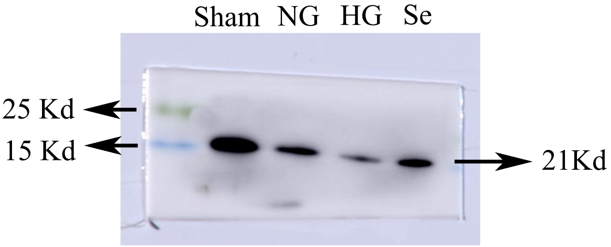

Fig 8. FT

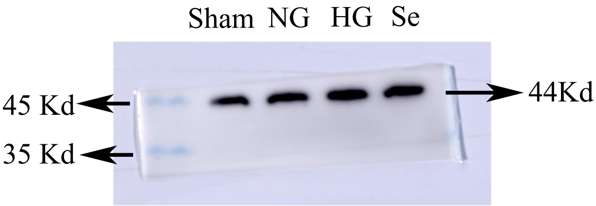

Fig 8. β- actin

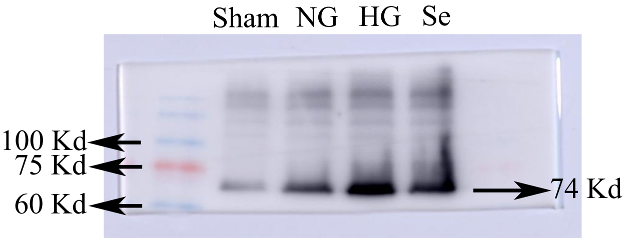

Fig 9. Ptgs2

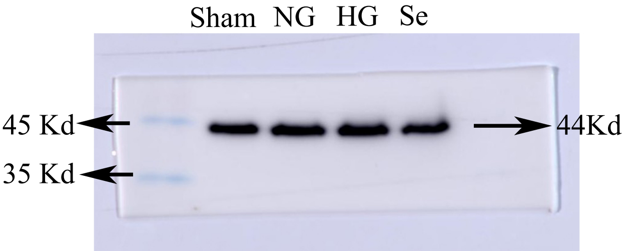

Fig 9. β- actin

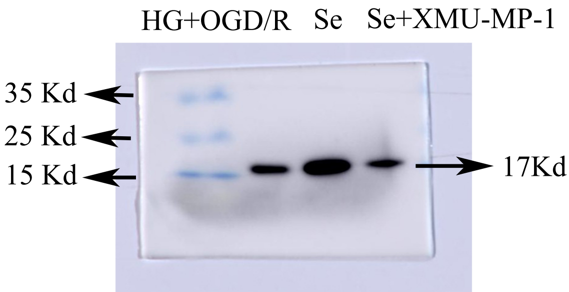

Fig 11. GPX4

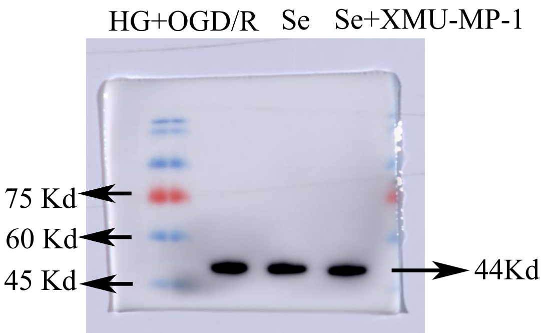

Fig 11. β- actin
